# Barriers and facilitators for participation in health promotion programs among employees: a six-month follow-up study

**DOI:** 10.1186/1471-2458-14-573

**Published:** 2014-06-09

**Authors:** Anne Rongen, Suzan JW Robroek, Wouter van Ginkel, Dennis Lindeboom, Bibiëlle Altink, Alex Burdorf

**Affiliations:** 1 Department of Public Health, Erasmus MC, University Medical Center Rotterdam, 3000 CA Rotterdam, The Netherlands; 2 Werkgeversforum Kroon op het Werk, 2132 JJ Hoofddorp, The Netherlands; 3 WerkVanNu, 2726 VA Zoetermeer, The Netherlands; 4 Lifeguard BV, 3508 AE Utrecht, The Netherlands

**Keywords:** Workplace health promotion, Participation, Barriers, Facilitators, Health behavior

## Abstract

**Background:**

Health promotion programs (HPPs) are thought to improve health behavior and health, and their effectiveness is increasingly being studied. However, participation in HPPs is usually modest and effect sizes are often small. This study aims to (1) gain insight into the degree of participation of employees in HPPs, and (2) identify factors among employees that are associated with both their intention to participate and actual participation in HPPs.

**Methods:**

Employees of two organizations were invited to participate in a six-month follow-up study (n = 744). Using questionnaires, information on participation in HPPs was collected in two categories: employees’ intention at baseline to participate and their actual participation in a HPP during the follow-up period. The following potential determinants were assessed at baseline: social-cognitive factors, perceived barriers and facilitators, beliefs about health at work, health behaviors, and self-perceived health. Logistic regression analyses, adjusted for demographics and organization, were used to examine associations between potential determinants and intention to participate, and to examine the effect of these determinants on actual participation during follow-up.

**Results:**

At baseline, 195 employees (26%) expressed a positive intention towards participation in a HPP. During six months of follow-up, 83 employees (11%) actually participated. Participants positively inclined at baseline to participate in a HPP were more likely to actually participate (OR = 3.02, 95% CI: 1.88-4.83). Privacy-related barriers, facilitators, beliefs about health at work, social-cognitive factors, and poor self-perceived health status were significantly associated with intention to participate. The odds of employees actually participating in a HPP were higher among participants who at baseline perceived participation to be expected by their colleagues and supervisor (OR = 2.87, 95% CI: 1.17-7.02) and among those who said they found participation important (OR = 2.81, 95% CI: 1.76-4.49).

**Conclusions:**

Participation in HPPs among employees is limited. Intention to participate predicted actual participation in a HPP after six months of follow-up. However, only 21% of employees with a positive intention actually participated during follow-up. Barriers, facilitators, beliefs about health at work, social-cognitive factors, and a poor self-perceived health status were associated with intention to participate, but hardly influenced actual participation during follow-up.

## Background

Companies increasingly offer workplace health promotion programs (WHPPs) to their employees. Poor health and unhealthy lifestyle are important causes of displacement from the labor force and productivity loss [1,2]. Workplaces are considered to be an effective setting for health promotion due to the possibility to reach a large proportion of the general population who spend a large amount of time there [3]. Hence, workplace health promotion programs have the potential to reach a large amount of persons aged 18 to 64, including many employees whose health and lifestyle needs improvement.

Systematic reviews have shown that WHPPs can improve lifestyle [4-7], increase productivity at work, and decrease sickness absence [8-11]. However, the effects of WHPPs are often small [12] and participation is usually modest [13], despite the fact that most employees are positive about health promotion at work [14,15]. Since small effects and low participation greatly diminish the potential gains of WHPPs [10], it is important to study the factors that potentially impede or facilitate participation.

Intervention studies are mainly concerned with studying the effectiveness of WHPPs. However, since low participation results in low effectiveness and is not cost-effective, it may just be as important to study participation. Participation is one of the aspects studied in process evaluations, which looks at reasons for success or failure of the program. However, such evaluations are often not conducted [16] and are often only used to evaluate newly developed WHPPs. In companies, the health promotion programs (HPPs) offered to employees might differ from those. Therefore, it is relevant to investigate determinants of participation among employees in companies that already offer HPPs that are aimed at changing various types of employees’ health behavior.

It is widely known that there are both barriers and facilitators to participation in a HPP. A review of the literature reveals multiple barriers that have claimed to impede participation HPPs. These include lack of time, lack of motivation, unfavorable work schedule, inconvenient location, costs, and already feeling healthy [17-21]. There is also evidence for facilitators, such as willingness to change one’s lifestyle [18,22]. However, there is a lack of studies investigating the extent to which these barriers and facilitators influence actual participation in WHPPs [13].

Social cognitive theories such as the Attitude Social-influence Self-efficacy (ASE) model [23] and the Theory of Planned Behavior (TPB) [24]) are often used when developing interventions for health promotion [25]. These theories identify intention as being a core construct that precedes actual behavior, and intention towards a behavior (e.g. intention to participate in a WHPP) is often measured as a proxy for actual behavior [26]. However, there is increasing debate regarding the gap between intention and actual behavior, a debate that addresses the issue of a positive intention not necessarily resulting in a behavior change [27,28]. It is therefore crucial to investigate both intention and actual behavior to become engaged in health promotion.

Due to the potential gain in health and work productivity and due to the positive attitude of employees to workplace health promotion, there is a clear need to investigate how participation can be increased. This study aims to (1) gain insight into the degree of participation of employees in HPPs, and (2) identify factors among employees that are associated with both their intention to participate and actual participation in HPPs.

## Method

### Study population

The population in this six-month follow-up study consisted of employees of a plastics manufacturer (organization 1, n = 874) and a paint manufacturer (organization 2, n = 1281) who held various jobs (e.g. office, laboratory, and manual workers). Both organizations had in place a variety of HPPs that were accessible for all employees. The organizations provided access to a fitness center either on site or close to the organization, consults with a dietitian and an occupational physician, smoking cessation programs, and mindfulness training. Policy changes were not considered as HPPs.

Between 2010 and 2012, all employees were invited by e-mail to fill in two online questionnaires: a baseline questionnaire and a follow-up questionnaire six months later. For this study, we included all employees who completed both the baseline and follow-up questionnaires.

Of the 2155 employees invited, 1128 (52%) completed the baseline questionnaire. Of this group, 761 (68%) also completed the follow-up questionnaire after six months and 748 employees (98%) provided informed consent. Four employees were excluded due to implausible or missing data on height, weight, or physical activity. The final study sample comprised 744 employees (organization 1, n = 279; organization 2, n = 465).

Informed consent was requested at the start of the baseline-questionnaire. The Medical Ethical Committee of Erasmus MC (Rotterdam, the Netherlands) declared that the Medical Research Involving Human Subjects Act did not apply to the current study and the committee had no objection to the execution of this study.

### Data collection

#### Intention to participate and actual participation

Three measures of participation in a HPP were assessed: intention to participate, actual participation before the start of the study, and actual participation during the six-month follow-up period. A HPP was defined in the questionnaire as follows: “A program that is aimed at improving your health behavior. For example, smoking cessation programs, fitness participation, participating in meetings on healthy nutrition.”

At baseline, participants were asked whether they had the intention of participating in a WHPP. To enhance comparability with actual participation, the five possible answers were dichotomized into ‘totally agree, agree’ and ‘totally disagree, disagree, neutral’.

At baseline, actual participation in a WHPP prior to enrollment in the study was assessed by asking participants whether they had participated in a WHPP in the past 12 months, and if so, what the topic of the program was (physical activity, healthy nutrition, smoking cessation, stress management, or health risks). Employees who had participated in multiple programs were asked to answer the question with regard to the most recent program followed.

At six-month follow-up, employees were asked whether they had participated in a HPP during the follow-up period. Employees who had participated were asked to name the topic of the HPP (physical activity, healthy nutrition, smoking cessation, stress management, or health risks), whereby multiple answers were permitted (i.e. multiple HPPs). For each topic, employees were then asked whether the HPP was organized through work or at their own discretion. Employees were classified as ‘sustainers’ if they had participated in a HPP in the year before enrollment and during the six-month follow-up period; as ‘new’ if they had not participated in the year before enrollment but had started a HPP during the follow-up period; and as ‘quitters’ if they had only participated in a HPP in the year before enrollment in the study.

### Social-cognitive factors

We formulated six statements that addressed attitude (two items i.e. importance of participating in WHPP, pleasantness of participating in WHPP), social support (three items i.e. support for participating in WHPP from supervisor, from colleagues, from friends and or family), and self-efficacy (one item i.e. believing that when willing to one succeeds in participating in a WHPP). The statements on support from supervisor and colleagues were combined into a single item (‘colleagues and or supervisor stimulate participation’ (Spearman’s Rho: 0.42)) that was positive when one of the underlying items was answered positively. The statements were based on important constructs from the Attitude-Social influence-Self-efficacy model [23] and were not strongly correlated (Spearman’s Rho range: 0.02-0.32). Since the purpose was to investigate whether the presence or absence of a factor was associated with participation, the five possible answers were dichotomized into ‘totally disagree, disagree, neutral’ and ‘totally agree, agree’.

### Barriers and facilitators

Employees were asked to indicate the degree to which potential barriers or facilitators would respectively impede or facilitate them in their decision to participate in a WHPP. We formulated two privacy-related barriers (e.g. ‘I would rather keep my work and private life separate’), two health-related barriers (e.g. ‘I’m healthy’), and another two work-related barriers (e.g. ‘I have an unfavorable work schedule’). Two health-related facilitators were formulated (e.g. ‘I want to improve my health’), and another two - work-related facilitators (e.g. ‘I find it enjoyable to work on my health together with colleagues’). Since the purpose was to investigate whether the presence or absence of a factor was associated with participation, the five possible answers were dichotomized into ‘totally disagree, disagree, neutral’ and ‘totally agree, agree’. Additionally, sum scores were calculated for barriers and facilitators based on the number of barriers and facilitators identified.

### Beliefs about health at work

Three statements were formulated that addressed employees’ beliefs with regard to workplace health promotion (e.g. ‘It is a good thing that my employer is trying to improve employees’ health’). Since the purpose was to investigate whether the presence or absence of a factor was associated with participation, the five possible answers were dichotomized into ‘totally disagree, disagree, neutral’ and ‘totally agree, agree’.

#### Self-perceived health and health behavior

Self-perceived health was measured using the first question of the Short Form-12 (SF-12) questionnaire (“Overall, how would you rate your health during the past 4 weeks?”). The five possible answers were dichotomized into ‘poor or fair’ and ‘good, very good, or excellent’ [29].

Body Mass Index (BMI: weight/height^2^) was calculated based on self-reported weight in kilograms and height in meters and categorized into normal weight (BMI < 25 kg/m^2^), overweight (25 ≤ BMI < 30 kg/m^2^), and obese (BMI ≥ 30 kg/m^2^).

Fruit and vegetable intake was measured using a slightly adapted version of the Dutch Food Frequency Questionnaire [30]. The six-item questionnaire asked about the monthly intake of different fruits (4 items, e.g. apple, fruit juice) and vegetables (2 items: cooked and raw vegetables). Dichotomization was based on the Dutch guidelines for healthy nutrition, which states that one should consume 200 grams of fruit and 200 grams vegetables daily. Employees who ate at least 400 grams of fruit and vegetables per day were considered those meeting the guidelines.

Physical activity was measured by a slightly adapted version of the International Physical Activity Questionnaire (IPAQ) [31], which measures physical activity of moderate and vigorous intensity. The average amount of leisure time spent on moderate and vigorous intensity physical activity was calculated as follows: employees were first asked how many days per week they engaged in moderate and vigorous intensity physical activity; they were then asked how many minutes on average was spent on moderate or vigorous intensity physical activity, per occasion. Dichotomization was based on recommendations for moderate intensity physical activity that requires such levels of activity for at least 30 minutes per day [32]. Employees who were physically active at a moderate intensity level for at least 210 minutes a week (7 times 30 minutes) were considered to have met this recommendation. Someone who was active at vigorous intensity for at least 20 minutes on at least three occasions per week met the recommendations for vigorous intensity physical activity.

Smoking was assessed using a single-item question: “Do you smoke?”. Answer possibilities were: ‘yes’, ‘now and then’, and ‘no’. Employees answering the question with ‘yes’ or ‘now and then’ were defined as being a ‘current smoker’.

#### Individual characteristics

The following individual characteristics were assessed: age, gender, and educational level. Age was categorized into three groups: 18–39, 40–49, 50–65. Educational level was determined by asking the employees about their highest level of education, which was then categorized at follows: low (primary school, lower and intermediate-level secondary schooling, or lower vocational training); intermediate (higher-level secondary schooling or intermediate vocational training); and high (higher vocational training or university).

### Data analysis

Descriptive statistics were used to report on the following: characteristics of the study population; participation prior to enrollment and during follow-up; barriers, facilitators, beliefs about health at work, and social-cognitive factors; and positive intention and actual participation according to number of barriers or facilitators perceived.

Logistic regression analyses, adjusted for age, gender, educational level, and organization, were used to study associations between the independent and dependent variables. The independent variables were barriers and facilitators, beliefs about health at work, social-cognitive factors, health behaviors, and self-perceived health. The dependent variables were intention to participate and actual participation during the six-month follow-up period.

Additional analyses were conducted to investigate whether the associations between health behaviors and self-perceived health on the one hand, and intention to participate and actual participation on the other, remained after adjustment for barriers, facilitators, moral beliefs, and social-cognitive factors. We also investigated whether selective loss to follow-up occurred.

The odds ratio (OR) was estimated as measure of association with a corresponding 95% confidence interval (95% CI). All analyses were carried out using the IBM SPSS Statistics version 20 for Windows (SPSS Inc., Chicago, IL, USA).

## Results

### Description of the study population

The study population consisted of 744 employees with a mean age of 44.9 years (SD: 9.2) and mean BMI of 25.7 kg/m^2^ (SD: 3.6). Further details are presented in Table 1.

**Table 1:** Characteristics of the study population (n = 744)

	**n**	**%**
**Individual characteristics**		
Age		
18-39	217	29.2
40-49	270	36.3
50-65	257	34.5
Male	548	73.7
Educational level		
Low	145	19.5
Intermediate	201	27.0
High	398	53.5
**Health behaviors and health**		
Body mass index		
Normal weight (BMI < 25 kg/m^2^)	359	48.3
Overweight (25 ≤ BMI < 30 kg/m^2^)	300	40.3
Obese (BMI 30 kg/m^2^ and higher)	85	11.4
Insufficient moderate physical activity (less than 30 min a day)	374	50.3
Insufficient vigorous physical activity (less than 3 days a week 20 min)	570	76.6
Insufficient fruit and vegetable intake (less than 400 grams a day)	493	66.3
Current smoker	140	18.8
Less than good self-perceived health	33	4.4
**Participation in a health promotion program**		
Intention to participate	195	26.2
Participated during the 12 month period prior to enrollment	95	12.8
Participation during the six-month follow-up period	83	11.2

The percentage of employees aged 50 years or older was higher in the group who completed both questionnaires than in the group who completed only the baseline questionnaire (34% versus 26%), but gender and educational level distribution were similar. Employees lost to follow-up did not differ from those completing both questionnaires with regard to their intention to participate in a WHPP, past participation, health behavior, or self-perceived health. However, the percentage of employees with high self-efficacy was significantly lower among employees lost to follow-up (51% versus 63%) and a higher percentage of this group reported the barrier ‘unfavorable work schedule’ (17% versus 12%) (data not shown).

### Participation in health promotion program

In the year before the baseline-measurement, 95 employees (13%) had participated in a WHPP (Table 2). During the six-month follow-up period, 83 employees (11%) participated in at least one HPP. The 83 employees participated in a total of 117 programs. Most employees participated in programs that were aimed at healthy nutrition (34%), health risks (32%), or physical activity (21%) (Table 2).

**Table 2:** Actual participation in a health promotion program before enrollment and during follow-up divided by topic

	**Participation before enrollment**	**Participation during follow-up**	**Participation sustainers, new, and quitters**		
			**Sustainers**	**New**	**Quitters**
	**n = 95**	**n = 83**	**n = 32**	**n = 51**	**n = 63**
Physical activity	33%	21%	20%	21%	35%
Healthy nutrition	40%	34%	34%	34%	37%
Smoking cessation	1%	4%	2%	5%	0%
Stress management	13%	9%	14%	7%	14%
Health risks	14%	32%	30%	33%	14%

Participation before enrollment: participation in a WHPP during the 12-month period prior to the baseline measurement. Participation during follow-up: participation in a HPP during the six-month follow-up period. Sustainers: employees who participated in a program both before enrollment and also during follow-up. New: employees who only participated in a program during follow-up. Quitters: employees who only participated in a program before enrollment.

During the six-month follow-up period, 32 employees (34%) had continued with at least one program after enrollment (sustainers), 51 employees (8%) started with at least one program during follow-up (new), and 63 employees (66%) quit following a program (quitters) (Table 2).

### Social-cognitive factors

At baseline, 195 employees (26%) had a positive intention towards participating in a WHPP. Of those, 40 employees (21%) actually participated in a program during the six-month follow-up period. Employees with a positive intention at baseline were more likely to actually participate during follow-up (OR = 3.02, 95% CI: 1.88-4.83) (Table 3).

**Table 3:** Characteristics of the determinants and their association with intention to participate and actual participation during follow-up

	**Positive on statement**	**Positive intention (n = 195)**	**Actual participation during follow-up (n = 83)**
	**n (%)**	**OR (95% CI)**	**OR (95% CI)**
Intention to participate in a WHPP	195 (26.2)	n/a	3.02 (1.88-4.83)*
Participated during the 12 month period prior to enrollment	95 (12.8)	5.92 (3.70-9.49)*	5.82 (3.40-9.96)*
**Social-cognitive factors**			
*Attitude*			
Important to participate	215 (28.9)	43.00 (26.83-68.91)*	2.81 (1.76-4.49)*
Pleasant to participate	620 (83.3)	8.64 (3.73-20.06)*	1.99 (0.93-4.27)
*Social support*			
Colleagues and or supervisor stimulate participation	68 (9.1)	2.83 (1.70-4.73)*	1.77 (0.90-3.49)
Family and or friends stimulate participation	79 (10.6)	6.84 (4.13-11.31)*	1.64 (0.86-3.15)
*Self-efficacy*			
High self-efficacy	467 (62.8)	4.43 (2.89-6.79)*	1.60 (0.96-2.66)
**Barriers**			
*Privacy related*			
Holding work and private preferably separate	371 (49.9)	0.44 (0.31-0.62)*	0.91 (0.57-1.46)
Want to organize it self	434 (58.3)	0.25 (0.18-0.36)*	0.92 (0.58-1.48)
*Health related*			
I’m healthy	531 (71.4)	0.74 (0.52-1.06)	1.25 (0.74-2.11)
Currently under treatment	140 (18.8)	1.32 (0.88-1.99)	1.50 (0.88-2.58)
*Work related*			
Unfavorable work schedule	90 (12.1)	1.48 (0.91-2.41)	0.64 (0.28-1.45)
Not knowing who to go to	77 (10.3)	1.67 (1.01-2.76)*	0.65 (0.27-1.56)
**Facilitators**			
*Health related*			
Wanting to improve my health	498 (66.9)	7.15 (4.26-12.00)*	1.44 (0.86-2.42)
Thinking a WHPP is useful	419 (56.3)	13.50 (7.98-22.83)*	1.45 (0.90-2.35)
*Work related*			
Pleasant to engage in activities with colleagues	150 (20.2)	3.78 (2.58-5.55)*	1.07 (0.61-1.89)
Supervisor or colleagues expect me to participate	28 (3.8)	3.00 (1.40-6.46)*	2.87 (1.17-7.02)*
**Beliefs about health at work**			
Good thing that the supervisor tries to improve employees health	599 (80.5)	4.44 (2.43-8.10)*	0.93 (0.52-1.65)
Interference of my supervisor on my health is an invasion of my privacy	139 (18.7)	0.45 (0.27-0.74)*	1.22 (0.69-2.17)
My health is a personal matter	485 (65.2)	0.69 (0.49-0.98)*	0.81 (0.50-1.30)

Positive intention: employees with a positive intention towards participating in a WHPP. Participation during follow-up: employees who participated in a HPP during the six-month follow-up period. Determinants are categorized into social-cognitive factors, barriers, facilitators, and beliefs about health at work.

Analyses adjusted for age, gender, educational level, and organization.

*statistically significant at p < 0.05.

n/a: not applicable.

Employees who had a positive attitude towards WHPPs, a high level of social support, and a high level of self-efficacy had significantly higher odds of having a positive intention towards participating in a WHPP, and had slightly higher odds of actual participation during follow-up (Table 3). In particular, a positive attitude towards the importance of participating in a WHPP was strongly associated with a positive intention (OR = 43.00, 95% CI: 26.83-68.91) and was also statistically significantly associated with actual participation during the six-month follow-up period (OR = 2.81, 95% CI: 1.76-4.49) (Table 3).

### Barriers and facilitators

**Figure 1:**
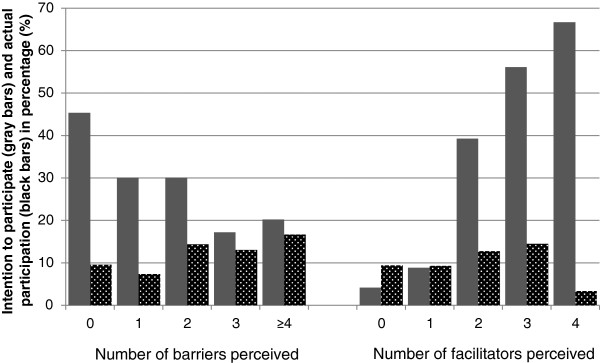
**Positive intention and actual participation according to the number of barriers and facilitators perceived.** The percentage of employees who had a positive intention (gray) and who actually participated (black) according to by the number of barriers (left) and facilitators (right) perceived by the employees.

The higher the number of barriers perceived by employees as preventing them from participating in a WHPP, the less likely they were to have a positive intention towards participating in a WHPP. The reverse pattern was observed for the number of facilitators perceived. These patterns were not observed for actual participation (Figure 1).

The most frequently mentioned barrier preventing participation in a WHPP was ‘I am already healthy’ (71.4%) and the most frequently mentioned facilitator was ‘I want to improve my health’ (66.9%) (Table 3).

Employees who stated that privacy-related factors would inhibit them from participating in a WHPP were more likely to have a negative intention towards participation. All facilitators increased the likelihood of having a positive intention towards participation (ORs: 3.00-13.50). An increased likelihood for actual participation was also observed for these barriers and facilitators, but to a lesser – non-significant - extent (Table 3).

### Beliefs about health at work

In total, 81% of participants thought it was a good idea that their employer would try to improve employees’ health, and only 19% considered it to be a violation of their privacy for their supervisor to interfere with their health. Employees who were positive about health promotion at work were more likely to have a positive intention towards participating in a WHPP, but were not more likely to actually participate (Table 3).

### Self-perceived health and health behaviors

Employees whose self-perceived health was less than good were more likely to have a positive intention towards participating in a WHPP (OR = 2.36, 95% CI: 1.15-4.82). However, such employees were not more likely to actually participate during follow-up (Table 4). None of the health behaviors were statistically significantly associated with either intention to participate or actual participation (Table 4). The strength of the associations between health and health behaviors and intention and participation barely changed following adjustment for barriers and facilitators (data not shown).

**Table 4:** Adjusted associations between health behaviors and self-perceived health, and positive intention and actual participation during follow-up

	**Positive intention (n = 195)**	**Actual participation during follow-up (n = 83)**
	**OR (95% CI)**	**OR (95% CI)**
**Health behaviors and health**		
Body mass index		
Overweight	1.00 (0.69-1.45)	1.21 (0.72-2.04)
Obesity	1.37 (0.80-2.33)	1.77 (0.89-3.58)
Insufficient moderate physical activity	0.87 (0.63-1.22)	1.56 (0.97-2.50)
Insufficient vigorous physical activity	0.72 (0.49-1.04)	0.71 (0.43-1.19)
Insufficient fruit and vegetable intake	0.71 (0.50-1.00)	1.16 (0.71-1.90)
Smoking	0.85 (0.55-1.31)	0.60 (0.30-1.18)
Less than good self-perceived health	2.36 (1.15-4.82)*	1.38 (0.51-3.71)

Positive intention: employees with a positive intention towards participating in a WHPP.

Actual participation during follow-up: employees who participated in a HPP during the six-month follow-up period.

Adjusted for age, gender, educational level, and organization.

*statistically significant at p <0.05.

## Discussion

A minority of the employees who responded (26%) had a positive intention towards participating in a WHPP, and even fewer employees (11%) actually participated during the six-month follow-up period. Although employees who had a positive intention were more likely to actually participate in a HPP, only 21% of those employees with a positive intention turned this into action by actually participating in a HPP. Employees who experienced barriers were more likely to have a negative intention while those who experienced facilitators were more likely to have a positive intention towards participating in a WHPP. Employees were also more likely to have a positive intention if they had a positive attitude towards WHPPs, a high level of social support, and a high level of self-efficacy and if their self-perceived health status was less than good. However, very few of the tested possible determinants predicted actual participation during the six-month follow-up period.

The fact that we found such low levels of participation is partly in line with findings from others. A systematic review has shown that participation varies greatly between WHPPs, with a median participation of 33% [13]. The fact that the studies included in this review targeted newly implemented programs, while the current study assessed participation in programs already offered by the organizations, might explain the lower levels of participation observed here. The organizations in our study did not implement any new HPPs during the study period. It is also possible that employees who were motivated to participate had already attended a program in the past and, therefore, did not participate again. This notion of newness improving participation is supported by the results of a Delphi study that found that exposure to a behavior change intervention improved when the content of the intervention was changed regularly [33].

Social cognitive theories such as the ASE-model [23] hypothesize that a positive attitude, high levels of social support, and high self-efficacy bring about a positive intention, which then leads to a behavior change. The first step is corroborated in this study: a positive attitude, a high level of social support, and a high self-efficacy were associated with a positive intention towards participation. However, our study could not corroborate the importance of specific behavioral determinants as observed in other studies on social-cognitive factors and actual behavior, for instance with an increase in fruit and vegetable intake [34,35]. Although the second step – from intention to behavior – is also supported by our results (i.e. a positive intention predicted actual participation), in absolute terms, only 21% of those with a positive intention actually participated. This corroborates the idea of the so-called intention–behavior gap, whereby a positive intention does not necessarily result in a behavior change. The modest proportion of 21% falls within the range of 18 to 60% observed in a meta-analysis that studied the relationship between intention and behavior with regard to physical activity [27]. The intention-behavior gap was also seen in two other meta-analyses, which demonstrated that, when implementing interventions, targeting intention has limited success in changing behavior [28,36]. In order to positively mediate the relationship of intention with behavior, careful planning, maintaining a high self-efficacy, and action control have been suggested [37]. So, although intention may predict behavior, researchers must be aware of a possible intention-behavior gap when conducting future research using intention as a proxy for behavior.

Almost all factors (i.e. social-cognitive factors, barrier, facilitators, and beliefs about health at work) were statistically significantly associated with intention to participate, but not with actual participation during the six-month follow-up period. This suggests that other factors play a role when deciding to actually participate. One explanation might be that programs do not match employees’ preferences. In other words, enrollment of participants may have been limited due to the set-up of the programs (e.g. group or individual programs; receiving information or completing assignments as content), the time at which the program takes place (e.g. after work hours) [20], or the way the program is delivered (e.g. provision of information, availability) [38]. A second reason might be the influence of the social environment on actual participation. Social ecological models hypothesize that an individual’s behavior is affected by factors at different levels: intrapersonal, interpersonal, institutional, community/society, and policy [39]. In this context, an employee might have the intention to participate in a program (intrapersonal), but may not be supported by management in executing his intended behavior (institutional level), for example in the case of WHPPs not being offered during work time [39]. Management support is found to be a major contributor to the success of WHPPs [40-42] which is supported by our results that showed that employees were more likely to participate when they felt that their supervisor or colleagues expects them to participate.

In an additional analysis, we found that barriers and facilitators had no influence on the transition from intention to participation. However, one should bear in mind that this analysis had limited power due to the small number of employees with a positive intention who also reported actual participation in a HPP.

Our finding that employees’ health behavior did not significantly influence their intention nor their actual participation during follow-up is in line with that of Groeneveld et al. [17]. Jorgensen et al. [43] described that employees with a moderate self-perceived health were more likely to contact a health professional. In our study, a low self-perceived health status was significantly associated with a positive intention, indicating that those employees who need it most are indeed interested. However, self-perceived health was not related to actual participation.

It has been suggested previously that research aimed at gaining more insight into the determinants of participation should focus on the underlying reasons for success and failure in participation [39]. Indeed, theories and frameworks such as the ‘Intervention mapping’ protocol [44] and participatory and peer-led interventions have been developed to this end, both aimed at developing successful interventions with a high take-up level by incorporating the needs and preferences of potential participants. Since the current study had an individualistic focus, future research needs to investigate the influence of the physical and social environment on actual behavior and whether this might partly explain the intention-behavior gap in participation.

### Limitations

This study has four main limitations. First, the fact that the study-design investigating associations with intention was cross-sectional does not permit further exploration with regard to causality. However, the relation between potential determinants and actual participation were studied using a study design with a six-month follow-up. Second, employees’ intention to participate was questioned about HPPs at the workplace, while actual participation was determined for HPPs both at the workplace and at employees’ own discretion. The data structure made it impossible to disentangle participation through work and at a private setting; employees could have indicated that they had participated in multiple programs, one of which might have been through work and the other in a private setting. Therefore, actual participation in programs organized or facilitated by the employer might be even lower. In addition, this discrepancy in how participation is questioned might have led to differences in ORs for intention and actual participation, since these factors relate to a greater extent to participation in a WHPP (intention) than to a HPP (actual participation). However, the relations with actual participation were not statistically significant. The third limitation stems from the low percentage of employees who actually participated in a HPP during the six-month follow-up period which resulted in a lack of statistical power. This is illustrated by several high non-significant ORs for relations between specific determinants and actual participation (e.g. ‘colleagues and or supervisor stimulate participation’). Future research in larger populations is recommended. Finally, selection bias as well as reporting bias cannot be ruled out. It could be hypothesized that employees with a low intention towards participating in a WHPP did not participate in this study. A large proportion of employees in this study had a negative intention towards participating in a WHPP therefore, this will most likely not have affected our results. However, the prevalence of a less than good self-perceived health was lower among participants (4.4%) than in the general Dutch population (19.9%) [45]. This difference might be partly explained by that the general population also includes unemployed and disabled persons who are more likely to have a poor self-perceived health status. For future research, it is recommended to gather also information on the health-related characteristics of non-responders. With regard to loss to follow-up, no differences were found with regard to gender, educational level, health, and health behaviors between employees who completed both questionnaires and those who completed only the baseline questionnaire.

## Conclusion

Overall, actual participation of employees in health promotion programs was limited. Although a positive intention predicted actual participation, most employees with a positive intention did not engage in a health promotion program during the six-month follow-up period, indicative of an intention-behavior gap. Employees with a positive attitude, high levels of social support, and a high self-efficacy were more likely to have a positive intention to participate in a WHPP. Employees perceiving barriers were less likely to express a positive intention towards participation, while the opposite was true of employees perceiving facilitators. Employees with a less than good self-perceived health status were more likely to have a positive intention, indicating that those employees who need it most are also those most interested. Actual participation was higher among those employees who considered participation important and thought it was expected of them by their supervisor or colleagues, corroborating the idea that the workplace could be a fruitful setting for health promotion.

## Competing interests

The authors declare that they have no competing interests.

## Authors’ contributions

DL, WG, and AB had the original idea for the study. AR, SR, and BA were responsible for data collection. AR carried out the data-analysis and drafted the manuscript. SR and AB provided methodological input. All authors participated in discussions and helped to draft the manuscript. All authors read and approved the final manuscript.

## Pre-publication history

The pre-publication history for this paper can be accessed here:

http://www.biomedcentral.com/1471-2458/14/573/prepub
